# Non-synergy of PD-1 blockade with T-cell therapy in solid tumors

**DOI:** 10.1136/jitc-2022-004906

**Published:** 2022-07-06

**Authors:** John S Davies, Farrah Karimipour, Ling Zhang, Nisha Nagarsheth, Scott Norberg, Carylinda Serna, Julius Strauss, Shinheng Chiou, James L Gulley, Christian S Hinrichs

**Affiliations:** 1 NCI, National Institutes of Health, Bethesda, Maryland, USA; 2 Safety Assessment, Genentech Inc, South San Francisco, California, USA; 3 Genitourinary Malignancies Branch, National Cancer Institute, Bethesda, Maryland, USA; 4 Immuno-Oncology Translational Medicine, AstraZeneca Gaithersburg, Gaithersburg, Maryland, USA; 5 Laboratory of Tumor Immunology and Biology, National Cancer Institute, Bethesda, Maryland, USA; 6 Cancer Immunotherapy, Rutgers Cancer Institute of New Jersey, New Brunswick, New Jersey, USA

**Keywords:** Costimulatory and Inhibitory T-Cell Receptors, CD8-Positive T-Lymphocytes, Combined Modality Therapy, Immunotherapy, Adoptive, Translational Medical Research

## Abstract

**Background:**

Cell therapy has shown promise in the treatment of certain solid tumors, but its efficacy may be limited by inhibition of therapeutic T cells by the programmed cell death protein-1 (PD-1) receptor. Clinical trials are testing cell therapy in combination with *PDCD1* disruption or PD-1-axis blockade. However, preclinical data to support these approaches and to guide the treatment design are lacking.

**Methods:**

Mechanisms of tumor regression and interaction between cell therapy and PD-1 blockade were investigated in congenic murine tumor models based on targeting established, solid tumors with T-cell receptor T cells directed against tumor-restricted, non-self antigens (ie, tumor neoantigens).

**Results:**

In solid tumor models of cell therapy, PD-1 blockade mediated a reproducible but non-synergistic increase in tumor regression following adoptive T-cell transfer. Tumor regression was associated with increased tumor infiltration by endogenous T cells but not by transferred T cells. The effect was independent of PD-1 receptor expression by transferred T cells and was dependent on the endogenous T-cell repertoire and on tumor antigenicity. PD-1 blockade primarily induced cell state changes in endogenous tumor-antigen-specific T cells rather than transferred T cells.

**Conclusions:**

Together, these findings support the concept that PD-1 blockade acts primarily through endogenous rather than transferred T cells to mediate a non-synergistic antitumor effect in solid tumor cell therapy. These findings have important implications for strategies to leverage PD-1 receptor disruption or blockade to enhance the efficacy of cell therapy.

**WHAT IS ALREADY KNOWN ON THIS TOPIC**

- Studies conducted primarily in models of chimeric antigen receptor (CAR)-T cell therapy in immunodeficient mice suggest that PD-1 blockade can enhance the efficacy of cell therapy. However, data from solid tumor models with intact endogenous T cell immunity and an intact PD-1–PD-L1 signaling axis are limited, and the mechanisms of interaction between cell therapy and PD-1 blockade are mostly unknown.

**WHAT THIS STUDY ADDS**

- This study reveals the non-synergistic effect of PD-1 blockade on T-cell therapy in solid tumors and elucidates a mechanism of action unexpectedly driven by endogenous rather than transferred T cells.

**HOW THIS STUDY MIGHT AFFECT RESEARCH, PRACTICE OR POLICY**

- These findings suggest that in cell therapy for immunogenic solid tumors PD-1 blockade may be a more effective strategy than PD-1 receptor knockdown or deletion and that PD-1-axis-targeting strategies may be relatively ineffective in non-immunogenic solid tumors. Furthermore, they raise the possibility that cell therapy and PD-1 blockade may be equally effective in combination or in sequence.

## Background

T-cell therapy is an emerging cancer treatment modality that holds promise for the treatment of solid tumors.^1 2^ Tumor infiltrating lymphocyte (TIL) therapy has demonstrated clinical activity in human papillomavirus (HPV)-associated cancers and melanoma.^3–5^ Genetically engineered T-cell receptor (TCR)-T cell therapy has shown tumor responses in a range of malignancies including cervical cancer, oropharyngeal cancer, vulvar cancer, anal cancer, melanoma, and synovial cell sarcoma.^6–8^ However, the efficacy of T-cell therapy in solid tumors has not matched that in hematologic cancers.^9^

In solid tumors, T-cell therapy may be limited in part by a hostile tumor microenvironment with constraint of T-cell effector function by immune checkpoint receptors.^10^ Programmed cell death protein-1 (PD-1) is an immune checkpoint receptor that is expressed by therapeutic T cells, host T cells, and other immune cells. It suppresses T-cell function by recruitment of phosphatases to an immunoreceptor tyrosine-based switch motif thereby countering positive signaling by the TCR and CD28.^11^ PD-1 also inhibits T-cell function by increasing expression of transcription factors such as basic leucine zipper transcriptional factor ATF-like, which inhibits T-cell effector function.^12^ Its principal ligand is programmed death-ligand 1 (PD-L1), which is expressed by tumors and tumor-infiltrating immune cells. Blockade of PD-1 or PD-L1 by systemic administration of monoclonal antibodies is an effective treatment for a wide-range of solid tumors.^13^

PD-1 inhibition in combination with cell therapy is an area of intense study in clinical trials. Preclinical data for the strategy derive mostly from murine models of chimeric antigen receptor (CAR)-T cell therapy including studies of HER2, PSMA, GD2, and CD19 CAR-T cells.^14–18^ One study demonstrated a modest improvement of tumor treatment when combining anti-PD-1 with anti-mesothelin CAR-T in NSG mice, which merits further investigation.^19^ Studies utilizing TCR-T cell models has been limited, although a modest advantage was suggested by a study in an immunodeficient mouse model and in a TCR-transgenic self-antigen model.^20 21^ A clinical trial, although with small patient cohorts, of CAR-T cells targeting GD2 did not demonstrate increased tumor responses or enhanced CAR-T cell expansion or persistence with addition of PD-1 blockade.^22^ A clinical trial of T cells CRISPR-engineered to express a TCR against NY-ESO-1 and to delete *PDCD1* did not demonstrate objective clinical responses in any of the three patients who were treated.^23^ One clinical trial demonstrated a benefit to patients following mesothelin-targeted CAR-T cell therapy and subsequent treatment with pembrolizumab.^24^ However, this study did not investigate if the benefit of combining CAR-T cells with pembrolizumab was through direct enhancement of the CAR-T cells. In addition, they demonstrated a clonal expansion of endogenous T cells in these patients.

Given the strong rationale, active clinical investigation, and limited preclinical and mechanistic research, we sought to investigate the combination of cell therapy with PD-1 blockade in solid tumor models. We employed tumor models based on TCR-T cell therapy rather than CAR-T cell therapy because clinical activity of engineered T-cell therapy in solid tumors has been observed primarily with TCR-based strategies. In addition, the varied, synthetic signaling domains of different CARs could yield results specific to a given CAR construct. Because the target antigens in successful solid tumor cell therapies have been tumor-restricted antigens (eg, neoantigens, viral antigens, or cancer germline antigens), tumor models were based on tumor-restricted antigens.^6–8^ Finally, due to complex interactions of PD-1 blockade with therapeutic T cells and host cells, syngeneic mouse model systems with intact PD-1 and PD-L1 expression by therapeutic cells and host cells were utilized.

## Methods

### Experimental model and subject details

#### Mice

C57BL/6J (B6, Stock # 000664), B6.CD45.1 (Stock # 002014), B6.OTI (Stock # 003831), B6.P14 (Stock # 004694), B6.*Rag1*-KO (Stock # 002216), B6.*Trac*-KO (Stock # 002116), and B6.*Pdcd1*-KO (Stock # 028276) were purchased from The Jackson Laboratory. B6.CD45.1.OTI, B6.CD45.1.P14 and B6.CD45.1.OTI.*Pdcd1*-KO were bred in our facilities. The experiments used mice that were between 6–12 weeks old (typically 8 weeks old) and when applicable the experiments were balanced for age and sex across the experimental groups. All mice were bred and maintained in a specific-pathogen-free facility certified by the Association for Assessment and Accreditation of Laboratory Animal Care International, and the study was carried out in accordance with protocols approved by the NCI Animal Care and Use Committee.

#### Tumor cell lines

B16F10-mKate2 (B16-K), B16F10-mKate2-OVA (B16-K-OVA), B16F10-mKate2-gp33 (B16-K-gp33), B16F10-mKate2-OVA-mtLNGFR-P2A-PD-L1 (B16-K-OVA-PD-L1), B16F10-mtLNGFR-P2A-mUb(G76V)-OVA (B16-U-OVA), B16F10-mtLNGFR-P2A-mUb(G76V)-gp33 (B16-U-gp33), B16F10-mtLNGFR-P2A-mUb(G76V)-OVA-htLNGFR-P2A-mUb(G76V)-gp33 (B16-U-OVA-gp33) and B16F10-mKate2-OVA-mtLNGFR-P2A-mUb(G76V)-gp33 (B16-K-OVA-gp33) cell lines were generated in house by transducing a new aliquot of B16-F10 (ATCC CRL-6475) from American Type Culture Collection (ATCC) with retroviral vectors. The red fluorescent protein called mKate2 is a 26.1 kDa protein that was derived from *Entacmaea quadricolor* and it is a foreign immunogenic protein in mice. Some vectors utilized a non-immunogenic self-protein, murine truncated Low-affinity Nerve Growth Factor Receptor (mtLNGFR), to facilitate the ability to identify and sort transduced cells. One vector utilized human tLNGFR (htLNGFR) to facilitate the ability to identify and sort cells transduced with two non-fluorescent vectors. The cell lines expressing both OVA and gp33 as well as the cell lines constitutively expressing PD-L1 were transduced twice, once with each virus, to achieve the resulting cell lines. The EL4-mKate2-OVA (EL4-OVA) cell line was generated as described above from a new aliquot of EL4 (ATCC TIB-39) obtained from ATCC. The MC-38-mKate2-OVA (MC38-OVA) cell line was generated as described above from a new aliquot of MC-38 (Kerafast ENH204) obtained from Kerafast. The MOC2-mKate2-OVA (MOC2-OVA) cell line was generated as described above and the parental MOC2 cell line was kindly provided by Dr Clint Allen (National Institutes of Health, Bethesda, Maryland, USA). All cell lines were cultured in complete medium containing DMEM (Gibco), 10% FBS (GE Healthcare), 10 mM HEPES (Gibco), 2 mM GlutaMAX (Gibco), 100 IU/mL penicillin and 100 µg/mL streptomycin (Gibco), 1× non-essential amino acids (Gibco), and 50 µg/mL gentamicin (Gibco). All cell lines were grown in a humidified incubator with 5% CO_2_ at 37°C and were routinely tested for mycoplasma contamination.

## Method details

### Mouse lymphocyte isolation, activation and expansion

OVA-specific CD8^+^ T lymphocytes (aka, OT-I, OTI, OT-1 or OT1 cells) were obtained from B6.OTI, B6.CD45.1.OTI or B6.CD45.1.OTI.PD-1-KO splenocytes and gp33-specific CD8^+^ T lymphocytes (P14 cells) were obtained from B6.P14 or B6.CD45.1.P14 splenocytes. All murine T cells were cultured in complete medium containing RPMI (Gibco), 10% FBS (GE Healthcare), 10 mM HEPES (Gibco), 2 mM GlutaMAX (Gibco), 100 IU/mL penicillin and 100 µg/mL streptomycin (Gibco), 1× non-essential amino acids (Gibco), 50 µg/mL gentamicin (Gibco) and 55 µM 2-mercaptoethanol (Gibco).

For experiments which did not transduce the T cells, the OTI splenocytes were stimulated with 0.5 µM SIINFEKL peptide (GenScript) and the P14 splenocytes were stimulated with 0.5 µM KAVYNFATM peptide (GenScript) and plated at 3×10^6^/mL into 24-well plates. The next day, 1 mL of complete media supplemented with 60 IU/mL interleukin (IL)-2 (aldesleukin from Prometheus) was added to each well. Cells are split as needed and fed daily with complete media will contain 60 IU/mL IL-2 from this point forward.

For experiments where transduced T cells were necessary, the same splenocytes were plated at the same density into 24-well plates coated with plate-bound anti-CD3ɛ (clone 145–2 C11 from Bio X Cell) at 2 µg/mL and supplemented with soluble anti-CD28 (clone 37.51 from Bio X Cell) at 1 µg/mL. As with peptide stimulation above, 1 mL of complete medium containing 60 IU/mL IL-2 was added to each well the day following initial stimulation. On day 2, all cells were harvested, washed and plated in complete media containing 60 IU/mL IL-2 at 5×10^5^–1×10^6^ cells/mL into 24-well plates that have been coated with retronectin (Takara) at 20 µg/mL and absorbed with the respective retroviral vector. The following day the cells are fed with 1 mL of complete media containing 60 IU/mL IL-2. On day 4 after stimulation, the cells are removed from the retronectin coated plate and seeded at 1×10^6^ cells/mL into 24-well plates and split as needed. In all experiments both in vitro and in vivo, the resulting T cells were used on day 6 after initial stimulation.

#### In vivo tumor inoculation, adoptive T-cell transfer and treatment

For in vivo tumor treatment efficacy experiments, tumor cell lines were injected subcutaneously at 1×10^6^ cells in 50 µL of HBSS into the right flank of 6–10 week old C57BL/6J, B6.*Rag1*-KO, B6.*Trac*-KO, or B6.P14 mice on day −7. On day −1, the mice were subjected to a single-dose total body irradiation at 5.5 Gy and their tumor was measured with calipers by first measuring the longest edge of the tumor followed by measuring at a 90^o^ angle to the first measurement. Mice without established palpable tumors or mice with tumors resulting from poor injections were not included in the tumor cohorts. The remaining mice were randomized into the groups within the cohort.

Adoptive T-cell therapy experiments were initiated on day 0 by injecting 1×10^6^–1×10^7^ T cells into tumor bearing hosts via intraperitoneal injections in 200 µL of HBSS. Antibody treatment was also initiated on day 0 with the injection of 200 µg per mouse in 100 µL of HBSS via intraperitoneal injection. The antibody injections occurred a total of four times, with additional injections on days 2, 4 and 6. In some experiments, the antibody treatment was delayed, the four injections were initiated on day 6 and continued on days 8, 10 and 12. The anti-PD-1 antibody used was clone RMP1-14 in the InVivoPlus formulation from Bio X Cell (Cat # BP0273).

### Mouse TIL isolation

For TIL isolation, the tumors were carefully removed from the mice and placed into complete RPMI and kept on ice. Tumors were then quickly blotted and weighed to obtain their mass for calculation purposes later. Next the tumors were subjected to the tumor cell isolation kit (Miltenyi #130-110-187) per manufacturer’s instructions and processed on the GentleMACS machine (Miltenyi #130-093-235). The required incubation steps were performed in cell culture incubators using the MACSmix Tube Rotators (Miltenyi #130-090-753). For flow cytometry experiments, the resulting single-cell preparation was negatively enriched for TIL by depleting red blood cells and a majority of tumor cells via magnetic depletion using anti-Ter119-biotin (Miltenyi #130-109-559) and anti-CD105-biotin (Miltenyi #130-118-107) followed by conjugating labeled cells to magnetic beads with anti-biotin magnetic beads (Miltenyi #130-097-046). For sequencing experiments, a debris removal step was included following manufacturer’s protocol (Miltenyi #130-109-398). Following this step, the Dead Cell Removal Kit (Miltenyi #130-090-101) protocol was combined with Pan T Cell Isolation Kit II (Miltenyi #130-095-130) and an additional 1 uL of anti-Ter119-biotin and anti-CD105-biotin were added to the cocktail of antibody. Twice the amount of anti-biotin magnetic beads were added, well within the acceptable range for the occupancy capacity of the paramagnetic isolation column. The final result for sequencing experiments was highly enriched, viable T cells with minimal contamination of debris and unwanted cell types.

### Cytotoxic killing assay

The in vitro cytotoxic killing assays were performed using an Agilent xCELLigence RTCA MP machine. Tumor targets were seeded at 10,000 cells per well the day prior to adding T cells. When applicable, the anti-PD-1 antibody (Bio X Cell #BP0273) or the isotype control anti-2A3 (Bio X Cell #BP0089) was added at the final concentration of 10 µg/mL just prior to adding T cells. When applicable, interferon (IFN)-γ (Peprotech #315–05) was added at a final concentration of 2 ng/mL the following morning after seeding the tumor cells, for 2 hours prior to adding the T cells. Then activated effector T cells on day 6 after in vitro activation, as described above, were added to the wells at the indicated effector-to-target ratios. The xCELLigence machine was set to measure impedance every 15 min throughout the duration of the experiment. Data were normalized to the time point immediately preceding the addition of effector T cells, per manufacturers recommendation.

#### In vitro proliferation and effector molecule production assays

For proliferation assay, as measured by CellTrace Violet, tumor targets were seeded into 48 well plates at 50,000 cells per well. The following day, day 6 activated effector T cells, as described above, were seeded into the plate at 1:1 effector-to-target ratio. Cells were stained as described in flow cytometry of mouse cells below. For effector molecule production assessment, tumor targets were seeded into 96 well plates at 20,000 cells per well. The following day, day 6 activated effector T cells, as described above, were seeded into the plate at 1:1 effector-to-target ratio. Cells were stained as described below in flow cytometry of mouse cells. The anti-PD-1 antibody and the anti-2A3 isotype control antibody (described above) were used at the final concentration of 10 µg/mL and the antibodies were added just prior to addition of effector T cells. The endpoint for the proliferation assay was day 4 after addition of T cells and the endpoint for the effector molecule induction was 24 hours after the addition of T cells.

### Defining reactivity of TCRs discovered in TIL

In order to identify the reactivity of the TCRs, we utilized a tumor panel that allowed us to determine which class of antigen(s) was the target in the event of observed reactivity. This panel was B16F10 (parental tumor, assessing pan-B16 antigens), B16-K (assessing mKate2 reactivity), B16-U-OVA (assessing SIINFEKL reactivity), B16-U-gp33 (assessing KAVYNFATM reactivity). If reactivity towards pan-B16 antigens was observed then there was an expectation of observing reactivity across all of the lines and if the reactivity was restricted to engineered antigens, then the reactivity was expected to be restricted to the line with the engineered antigen. These tumor targets were seeded into the in vitro cytotoxic killing assay as described above. The positive control T cells included OT-I and P14 T cells. TCRs identified from TIL single-cell RNA sequencing experiments were used to generate MSGV-based retroviral vectors in order to express these TCRs on polyclonal T cells (C57BL/6J splenocytes that were stimulated and transduced as described above). The top 14 most prevalent TCRs from untreated, OT-I treated, anti-PD-1 treated and OT-I + anti-PD-1 treated groups were selected as well as all shared TCRs between the groups (as determined at the amino acid level, as none were shared at the nucleotide level) regardless of the prevalence of the shared TCRs were assessed. In total, 69 different TCRs were assessed by this method.

### Mouse tumor immunofluorescence

Tumors were harvested on day 7 after treatment was initiated and placed in 10% formalin for 24 hours after which they were placed in 70% ethanol until processing. Tumor samples were embedded into paraffin blocks and slides were prepared. Slides were placed in 250 mL of citric acid buffer into a slide holding container. Heat buffer solution in microwave for 10 min to 100°C, allowed to cool and reheated to 100°C for 2 min. This process was repeated two times. The citric acid buffer was replaced with phosphate-buffered saline (PBS) and the slides were washed and subsequently blocked with bovine serum albumin. The anti-PD-L1 primary antibody (Cell Signaling Technology #13684) was used at 1:200 dilution in PBS and incubated with slides overnight at 4°C. Slides were washed twice with PBS and a third time with PBS with Tween 20 (PBST). The goat anti-rabbit FITC secondary antibody (Invitrogen #F-2765) was used at 1:1000 dilution in PBS and incubated with slides for 50 min at room temperature. Slides were washed twice with PBS and a third time with PBST. Sudan black was used for 5 min to block the background. Running water wash was performed for 20 min. Then immunofluorescent imaging of the coverslip was performed for the final endpoint.

### Flow cytometry of mouse cells

The following panel was used to assess PD-1 expression on in vitro activated OT-I cells: fixable viability dye eFluor 506 (eBioscience #65-0866-18), PE-labeled anti-Va2 TCR (BD #553289) and PE-Cy7-labeled anti-PD-1 (Invitrogen #25-9985-82). The following panel was used to assess proliferation of OT-I cells cocultured with tumor targets with or without anti-PD-1 blocking antibody: CellTrace Violet (Invitrogen #C34557) was used per manufacturers protocol to label OT-I cells and the cells were further isolated from tumor targets using PE-labeled anti-CD45 (Miltenyi #130-110-797) and dead cells excluded with Live-Dead NIR (Invitrogen #L10119). The following panel was used to assess effector molecule production by OT-I cells cocultured with tumor targets with or without anti-PD-1 blocking antibody: eFluor660-labeled anti-Granzyme B (Invitrogen #50-8898-82), BUV395-labeled anti-CD45 (BD #564279), BUV737-labeled anti-IFN-γ (BD #612769), fixable viability dye eFluor 506 (eBioscience #65-0866-18), VioBright515-labeled anti-Ki67 (Miltenyi #130-120-421), PE-labeled anti-TNFa (Invitrogen #12-7321-82), and PerCP-e710-labeled anti-4-1BB (Invitrogen #46-1371-82). Intracellular staining was achieved by using the BD Cytofix/Cytoperm kit (BD #554714). The following antibodies were used to assess PD-L1 and H-2K^b^ upregulation by B16F10 cells exposed to IFN-γ (Peprotech #315–05) at 2 ng/mL: PE-Cy7-labeled anti-PD-L1 (Invitrogen #25-5982-82) and BV421-labeled anti-H-2K^b^ (BD #562942). The following panel was used to assess TIL: VioBright515-labeled anti-CD45.1 (Miltenyi #130-121-222), BUV395-labeled anti-CD45.2 (BD #564616), BV421-labeled CX3CR1 (BD #149023), BV650-labeled anti-4-1BB (BD #740499), APC-labeled anti-TIM3 (Miltenyi #130-119-756), SuperBright 702-labeled anti-LAG3 (Thermo #67-2231-82), PE-Vio770-labeled anti-CD69 (Miltenyi #130-115-577), PE-CF594-labeled anti-CD103 (BD #565849), APC-Vio770-labeled anti-CD3e (Miltenyi #130-117-676), BUV737-labeled anti-CD8a (BD #564297), PerCP-Vio700-labeled anti-CD4 (Miltenyi #130-118-794), PE-labeled anti-PD-1 (BD #551892), SuperBright 600-labeled anti-PD-L1 (Thermo #63-5982-82), fixable viability dye eFluor 455UV (eBioscience #65-0868-18), additionally CountBright absolute counting beads (Invitrogen #C36950) was used in these experiments.

### Single-cell RNA-sequencing and single-cell TCR-sequencing data processing

Samples were obtained via as described above in TIL isolation. There were five tumors per group and each tumor was individually barcoded with 5’ total-seq cell hashing antibodies (BioLegend #155861, 155863, 155865, 155867, and 155869). Afterwards the samples were pooled and processed for single-cell RNA sequencing (RNA-seq) partitioning and library preparation.

Single-cell suspensions were washed twice with ice-cold PBS by centrifugation at 300 g and resuspended in fresh buffer. Cell counts and viability measurements were performed for each sample on a fluorescent cell counter and propidium iodide and acridine orange dyes (LunaFL, Logos Biosystem). After adjusting cell concentrations, samples were loaded onto the 10x Genomics Chromium platform using the 5’ v1.1 gene expression chemistry (PN-1000165) on Chromium Next GEM Chip G (PN-1000120) targeting 10,000 cells when sample amounts and viabilities allowed. Preparation of libraries were performed according to vendor recommendations using commercially available kits, including the generation of the 5’ RNA-Seq library (PN-1000020), the mouse TCR enrichment library (PN-1000071), and the feature barcode library (PN100080).

Single-cell RNA-Seq libraries were sequenced on an Illumina 550 instrument according to 10x Genomics recommendations. For gene expression libraries, a Read2 length of 98 base-pairs was used to identify complementary DNA. Associated TCR libraries were sequenced separately with paired-end read lengths of 150 bp to allow robust VDJ spanning coverage. Feature barcode libraries were also sequenced independently, with a Read2 length of 55 base-pairs to identify the feature barcode identity.

Raw sequencing data were processed using the 10x Genomics provided *cellranger* (V.3.1.0) pipeline to demultiplex data into fastqs, and then align reads to the provide mouse references (refdata-cellranger-mm10-3.0.0 for gene expression reads), (refdata-cellranger-vdj-GRCm38-alts-ensembl-3.1.) for TCR reads, and a custom list of the feature barcode sequences used in this experiment. This *cellranger* pipeline then generated single-cell expression matrices which allowed associating of TCR and feature barcode outputs with the gene expression data.

The single-cell samples were processed primarily using the R statistical program (V.3.6.1). Sample transcript and cell hashing counts were imported individually into the R environment using the Seurat package (V.3.1.4) with the ‘Read10X_h5’ function, followed by assigning each data matrix to separate data assays with the ‘CreateSeuratObject’ function initially with the transcript counts matrix and the ‘CreateAssayObject’ function with the cell hashing counts matrix. Preliminary quality control for identification and removal of heterotypic doublets was performed by first normalizing the cell hashing assay via the centered-log ratio transformation, using the ‘NormalizeData’ function and demultiplexing using the ‘HTODemux’ function, with the 99th percentile of counts used as a cut-off for positive identification. Subsequent quality control was performed using functions available in Seurat, unless otherwise specified. Initial cell quality was evaluated using the Routliers package, with cells being excluded under one of the following conditions: exceeding the lower limit of three median absolute deviations (MADs) for number of features (ie, unique genes); exceeding the lower limit of three MADs for number of total reads; or exceeding the upper limit of three MADs for fraction of reads being mitochondrial genes. Read count normalization and scaling was performed using the SCTransform algorithm with 3000 variable features, as determined with the ‘FindVariableFeatures’ function. Algorithmic doublet identification and removal was conducted using the DoubletFinder tool. Samples were combined and rescaled using the SCTransform-specific integration pathway, specifically with the functions ‘SelectIntegrationFeatures’, ‘PrepSCTIntegration’, and ‘IntegrateData’, the latter with the flag ‘normalization.method=SCT’.

The Marchenko-Pastur method, as implemented in the ‘URD’ package, was used to identify 50 principal components as significant. These principal components were then used to generate a Uniform Manifold Approximation Projection (UMAP) with the ‘RunUMAP’ function and perform shared nearest neighbor clustering using the Smart Local Moving algorithm with a resolution of 0.4, using the ‘FindNearestNeighbors’ and ‘FindClusters’ functions. Differentially expressed genes were identified using the ‘Model-based Analysis of Single-cell Transcriptomics’ algorithm as available in the ‘FindMarkers’ function. Pathway analysis at the single cell resolution was performed using the ‘AUCell’ package in R, and at the cluster resolution using single-sample Gene Set Enrichment Analysis (‘ssGSEA’) as available through the Gene Set Variation Analysis (‘GSVA’) package.

Additional V(D)J classification was deemed necessary as many of the results that came out of the 10x pipeline had more unique alpha chain and/or beta chain sequences than what is biologically possible for a single cell. Only for TCR analyses, we removed events that had more than two unique beta chain reads and more than two unique alpha chain reads, with the exception of events that were determined to have the OT-I TCR. We utilized OT-I mice that were Rag-sufficient, which caused us to filter events with OT-I TCRs in a manner that required a productive OT-I TCR, did not allow for any productive beta chains and allowed for up to two unique alpha chain sequences (regardless if productive or not). All of these analyses were done at the nucleotide level for the sequences.

The GLIPH2 algorithm was used to analyze TCRβ chains from the single-cell TCR sequencing data using default settings in software.

## Results

### PD-1 blockade enhanced adoptive T-cell therapy and was associated with decreased tumor infiltration by transferred T cells and increased tumor infiltration by endogenous T cells

**Figure 1:**
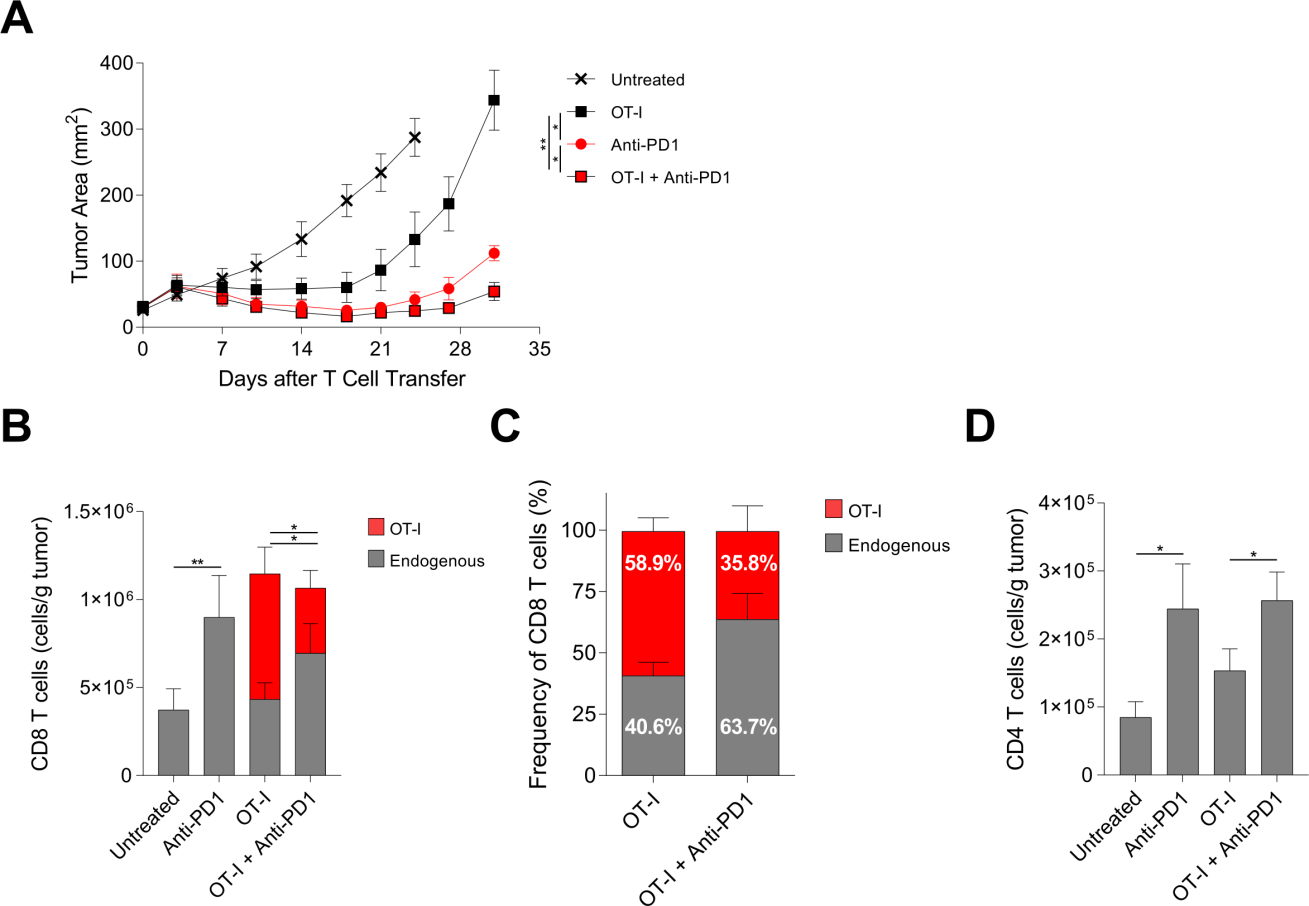
PD-1 blockade enhanced adoptive T-cell therapy and was associated with decreased tumor infiltration by transferred T cells and increased tumor infiltration by endogenous T cells. (A) Tumor response to treatment with adoptive T-cell therapy with or without PD-1 blockade. C57BL/6J (CD45.2^+^) mice with established B16-K-OVA tumors were treated with host conditioning followed by the treatment indicated in the symbol legend. ‘OT-I’ indicates OT-I T cell (CD45.1^+^) administration. ‘Anti-PD-1’ indicates anti-PD-1 antibody administration. Statistical significance was determined with a mixed model two-way analysis of variance with repeated measures. The data shown are representative of three independent experiments. (B) Absolute numbers (cells/g tumor) of transferred OT-I T cells (CD45.1^+^, red) and endogenous CD8 T cells (CD45.2^+^, gray) infiltrating tumors on day 7 after treatment. Statistical significance was determined with an unpaired t-test. (C) Proportion of transferred OT-I T cells (CD45.1^+^, red) and endogenous CD8 T cells (CD45.2^+^, gray) infiltrating tumors on day 7 after adoptive cell transfer. (D) Absolute numbers (cells/g tumor) of endogenous CD4^+^ T cells infiltrating tumors on day 7 after adoptive cell transfer. Statistical significance was determined with an unpaired t-test. All data have five mice per group and are represented as mean±SEM. * represents p<0.05, ** represents p<0.01. See also online supplemental figure S1. PD-1, programmed cell death protein-1.

To test if PD-1 blockade increased the efficacy of solid tumor TCR-T cell therapy in a murine model, B16F10 melanoma tumors with stable expression of a mKate2-SIINFEKL fusion protein (B16-K-OVA) were treated with H-2K^b^:SIINFEKL-specific OT-I TCR transgenic T cells. In this model, transferred tumor-infiltrating OT-I T cells demonstrate high PD-1 expression and B16-K-OVA tumors demonstrate strong PD-L1 expression in vivo, indicating an intact PD-1-PD-L1 axis (online supplemental figure 1A). Additional phenotyping of activating markers and inhibitory receptors of TIL indicated that transferred OT-I T cells exhibited increased expression of 4-1BB, LAG3, PD-1, PD-L1, and CD69 with only TIM3 increased on endogenous CD8 TIL (online supplemental figure 1C). Treatment with either adoptive transfer of OT-I T cells or with PD-1 blockade alone showed tumor regression (there was experimental variability as to which single-agent modality provided the greater treatment effect) (figure 1A). Notably, addition of PD-1 blockade to OT-I T cell therapy consistently increased treatment efficacy (figure 1A). To determine if the increased efficacy from addition of PD-1 blockade to T-cell therapy was associated with increased tumor infiltration by transferred tumor-targeting T cells, the concentrations of tumor infiltrating OT-I T cells and endogenous CD8 T cells after treatment were determined (figure 1B). Addition of PD-1 blockade increased the concentration of tumor-infiltrating endogenous CD8 T cells but decreased the concentration of tumor-infiltrating OT-I T cells (figure 1B). This decrease in OT-I T cells with a reciprocal increase in endogenous T cells was also reflected in the frequency of tumor-infiltrating CD8 T cells from each compartment (figure 1C). Addition of PD-1 blockade to OT-I T cell therapy also increased tumor infiltration by endogenous CD4 T cells (figure 1D). These findings indicated that PD-1 blockade improved tumor treatment while increasing tumor infiltration by endogenous T cells and decreasing tumor infiltration by transferred T cells.

10.1136/jitc-2022-004906.supp1 Supplementary data

### OT-I T cell antitumor function was not improved by PD-1 blockade in in vitro assays

**Figure 2:**
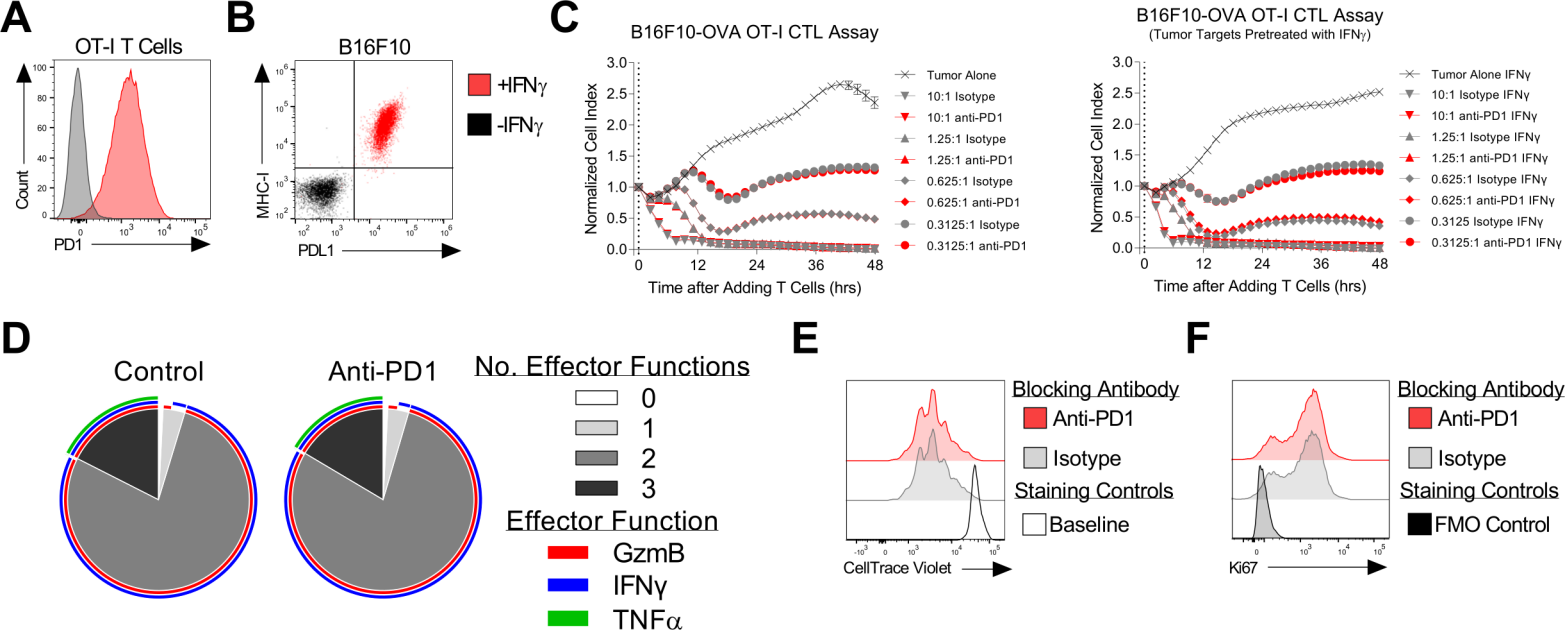
OT-I T cell antitumor function was not improved by PD-1 blockade in in vitro assays. (A) Expression of PD-1 by OT-I T cells as determined by flow cytometry before (gray) and after (red) activation and expansion in vitro. (B) Expression of MHC-I (H-2K^b^) and PD-L1 by the B16F10 tumor cell line as determined by flow cytometry either with (red) or without (black) pretreatment with IFN-γ. (C) OT-I T cell-mediate cytolysis of B16F10-K-OVA tumor cells with anti-PD-1 antibody blockade (red) or isotype antibody (gray) as assessed by real-time impedance-based killing assay. ‘Tumor Alone’ is a negative control of tumor cells without OT-I T cells. The effector to target ratio is shown on each graph. The experiments was performed with (right panels) or without (left panels) pretreatment of tumor cells with IFN-γ. The mean±SEM is graphed. The data shown are representative of three independent experiments. (D) OT-I T cell polyfunctionality with (right, ‘anti-PD-1’)) and without (left, ‘control’) anti-PD-1 antibody blockade as determined by intracellular flow cytometric analysis of effector molecule expression. Sunburst plots show data from 24 hours after coculture with B16-K-OVA tumors. The outer rings indicate specific effector molecules. The inner pie chart indicates the number of effector functions. The data shown are representative of two independent experiments. (E) Proliferation of OT-I T cells in response to B16-K-OVA tumors in the presence of the antibody indicated in the symbol legend. A CellTrace Violet dilution assay performed 4 days after coculture initiation is shown. The open gray histogram is the baseline labeling control. (F) Ki67 expression by OT-I T cells 24 hours after coculture with B16-K-OVA tumors with the antibody indicated in the symbol legend. GzmB, granzyme B; hrs, hours; IFN, interferon; PD-1, programmed cell death protein-1; PD-L1, programmed death-ligand 1; TNF, tumor necrosis factor.

We next investigated the direct impact of PD-1 blockade on the effector function of therapeutic OT-I T cells in vitro. In this in vitro system, therapeutic OT-I T cells displayed high expression of PD-1, which may be present as an activation marker in this setting, and the B16-K-OVA tumor cell line showed IFN-γ-inducible expression of PD-L1 (figure 2A and B). PD-1 blockade did not increase OT-I cytolysis of B16-K-OVA cells in a 48-hour real-time assay, even when PD-L1 expression was induced by pretreatment with IFN-γ (figure 2C). Expression of the effector molecules granzyme B, IFN-γ, and tumor necrosis factor-α was also not increased by PD-1 blockade (figure 1D). Finally, proliferation as assessed by cell division assay and by Ki67 expression assay was not increased by PD-1 blockade (figure 2E and F). Together these data indicated that PD-1 blockade did not directly enhance the antitumor effector function of therapeutic T cells in vitro, although in vitro conditions are not as relevant as the tumor microenvironment in vivo.

### Enhancement of cell therapy by PD-1 blockade was independent of transferred T cell *Pdcd1* expression and was dependent on endogenous T cells

**Figure 3:**
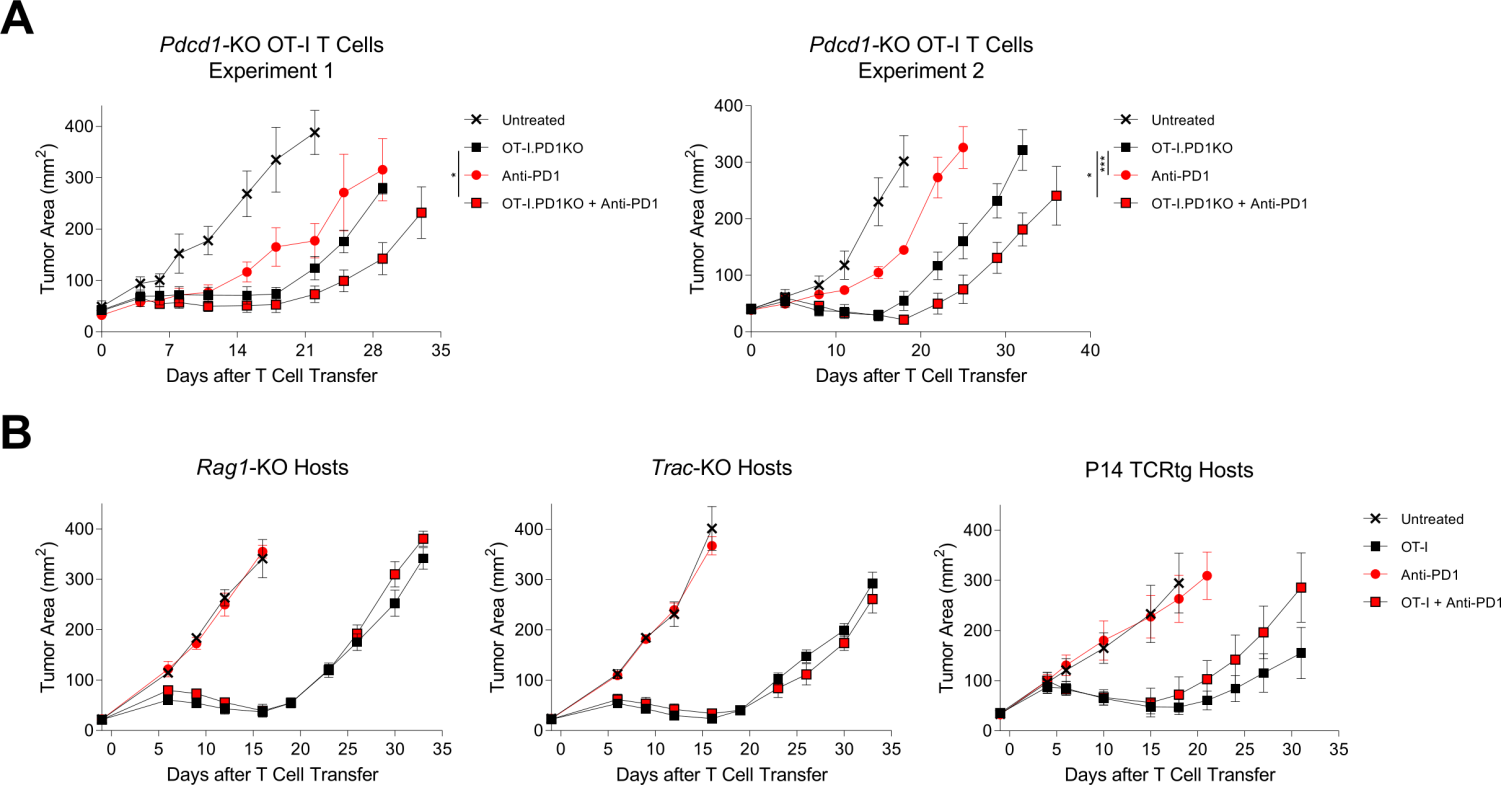
Enhancement of cell therapy by PD-1 blockade was independent of transferred T cell *Pdcd1* expression and was dependent on endogenous T cells. (A) Tumor curves showing treatment of C57BL/6J mice bearing established B16-K-OVA tumors with adoptive transfer of *Pdcd1*-KO OT-I T cells, with or without PD-1 blockade. Mice received host conditioning followed by the therapy indicated in the symbol legend. ‘OT-I.PD1KO’ indicates administration of *Pdcd1*-KO OT-I T cells. ‘Anti-PD-1’ indicates administration of anti-PD-1 antibody. Statistical significance was determined with a mixed model two-way analysis of variance with repeated measures. * represents p<0.05, *** represents p<0.001. Two independent experiments shown. (B) Tumor curves showing treatment of host mice with absent or restricted endogenous T-cell repertoires with wild-type OT-I T cells, with or without PD-1 blockade. The knockout gene or transgene for the host mice in each experiment is indicated above each tumor graph. The symbol legend shows the treatment that was given. ‘OT-I’ indicates OT-I T cell administration. ‘Anti-PD-1’ indicates anti-PD-1 antibody administration. The data shown have five mice per group and are representative of two independent experiments. Data are represented as mean±SEM. PD-1, programmed cell death protein-1.

To investigate the direct effect of PD-1 blockade on the transferred therapeutic T cells in vivo, mice with B16-K-OVA tumors were treated with PD-1 receptor deficient (*Pdcd1*^−/−^) OT-I T cells with or without PD-1 blockade (figure 3A). The efficacy of *Pdcd1^−/−^* OT-I cells was increased by PD-1 blockade indicating independence of the PD-1 blockade effect from direct interaction with the PD-1 receptor of transferred T cells. To further investigate the interaction of PD-1 blockade with transferred versus endogenous T cells, we studied treatment of T cell-deficient host mice. Treatment of B16-K-OVA tumors was not enhanced by PD-1 blockade in *Rag1^−/−^* mice or in *Trac^−/−^* mice (figure 3B, left and middle). To determine if this lack of an effect was due to the absence of T cells, the treatment was also studied in P14 TCR transgenic host mice that are T cell-replete but have a restricted T-cell repertoire (figure 3B, right). PD-1 blockade also did not enhance the cell therapy treatment in this setting. These findings indicated that endogenous T cells were necessary for the enhanced therapeutic effect of PD-1 blockade in this solid tumor cell therapy model.

### PD-1 blockade did not enhance cell therapy in endogenous T-cell-deficient hosts in wide-ranging tumor models

**Figure 4:**
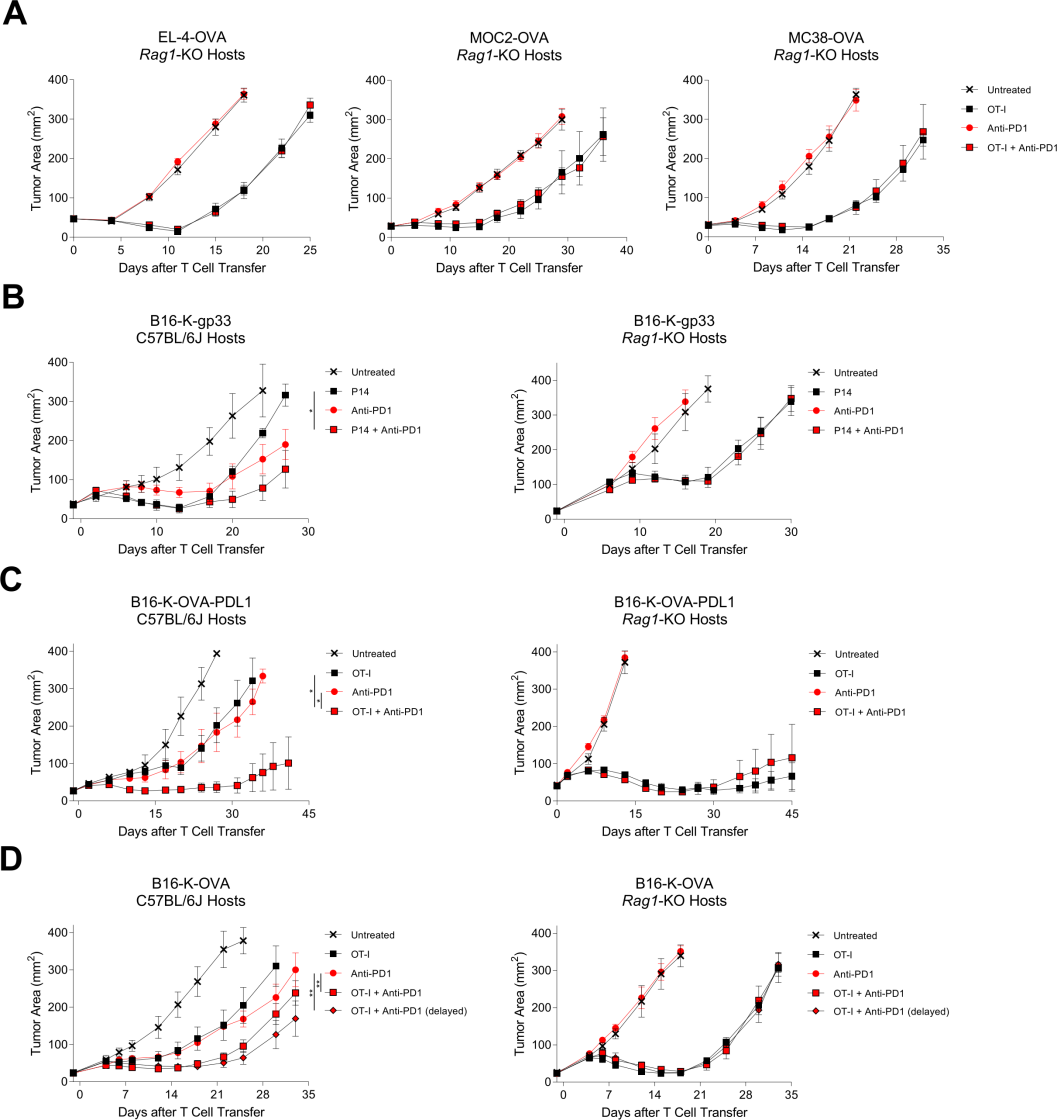
PD-1 blockade did not enhance cell therapy in T-cell deficient hosts in wide-ranging tumor models. Tumor curves demonstrating response to treatment with adoptive T-cell therapy, with or without PD-1 blockade across a range of parental tumors, target antigens, transferred T cells, and other conditions. The characteristics of the transferred T cells, tumors, and host mice are depicted to the left of each tumor graph. (A) *Rag1*-KO mice with established EL-4-OVA, MOC2-OVA, or MC38-OVA tumors were treated with the therapy indicated in the symbol legend. ‘OT-I’ indicates OT-I T cells. ‘Anti-PD-1’ indicates anti-PD-1 antibody. The data shown are representative of two independent experiments. (B) C57BL/6J wild-type (left) or *Rag1*-KO (right) mice with established B16-K-gp33 tumors were treated with P14 T cells, with or without PD-1 blockade. ‘P14’ indicates P14 T cells. ‘Anti-PD-1’ indicates anti-PD-1 antibody. The data shown are representative of three independent experiments. (C) C57BL/6J wild-type (left) or *Rag1*-KO (right) mice with established B16-K-OVA-PD-L1 (constitutive expression of PD-L1) tumors were treated with the therapy indicated in the symbol legend. ‘OT-I’ indicates OT-I T cells. ‘Anti-PD-1’ indicates anti-PD-1 antibody. The data shown are representative of three independent experiments. (D) C57BL/6J wild-type (left) or *Rag1*-KO (right) with established B16-K-OVA tumors were treated with the therapy indicated in the symbol legend. ‘OT-I’ indicates OT-I T cells. ‘Anti-PD-1’ indicates anti-PD-1 antibody following the standard treatment course (administration on days 0, 2, 4, and 6). ‘Anti-PD-1 (delayed)’ indicates anti-PD-1 antibody following a delayed treatment course (administration on days 6, 8, 10, and 12). The data shown are representative of three independent experiments. Statistical significance for all panels was determined with a mixed model two-way analysis of variance with repeated measures. Data for all panels have five mice per group and are represented as mean±SEM. * represents p<0.05, ** represents p<0.01. PD-1, programmed cell death protein-1

To investigate if the requirement for endogenous T cells was specific to treatment of B16-K-OVA tumors with OT-I TCR transgenic T cells, we systematically assessed different tumor and treatment models. First, we changed the tumor type to EL-4-OVA, MOC2-OVA, and MC38-OVA. PD-1 blockade did not enhance the antitumor effect of transferred T cells for any of these tumors (figure 4A). Next, we changed the target antigen and therapeutic TCR from H-2K^b^:SIINFEKL (OVA) and OT-I TCR to H-2D^b^:KAVYNFATM (gp33) and P14 TCR, respectively. As with the OT-I model, in the P14 model PD-1 blockade enhanced cell therapy in wild-type hosts, and the effect was lost in T cell-deficient hosts (figure 4B). Next, to exclude the possibility that PD-L1 expression by B16-K-OVA is insufficient to suppress OT-I T cells, tumors with constitutive, high levels of PD-L1 expression were generated (B16-K-OVA-PD-L1). Treatment of PD-L1-overexpressing tumors with cell therapy was enhanced by PD-1 blockade in wild-type hosts but not in T cell-deficient hosts (figure 4C). Finally, the anti-PD-1 antibody dosing regimen was changed to administration on days 6, 8, and 10. As with the initial regimen of dosing on days 2, 4 and 6, this regimen increased the efficacy in wild-type hosts but not *Rag*1^−/−^ hosts (figure 4D). Attempting to delay anti-PD-1 treatment further proved difficult due to the aggressive growth rate of B16 tumors and required animal care and use committee (ACUC) endpoint criteria. These results showed that improvement of cell therapy by PD-1 blockade was dependent on host T cells irrespective of the parental tumor, target antigen, tumor PD-L1 expression, or anti-PD-1-antibody timing.

### The effect of PD-1 blockade was dependent on tumor antigenicity and it was associated with changes in the cell state of tumor-antigen-specific endogenous T cells

**Figure 5:**
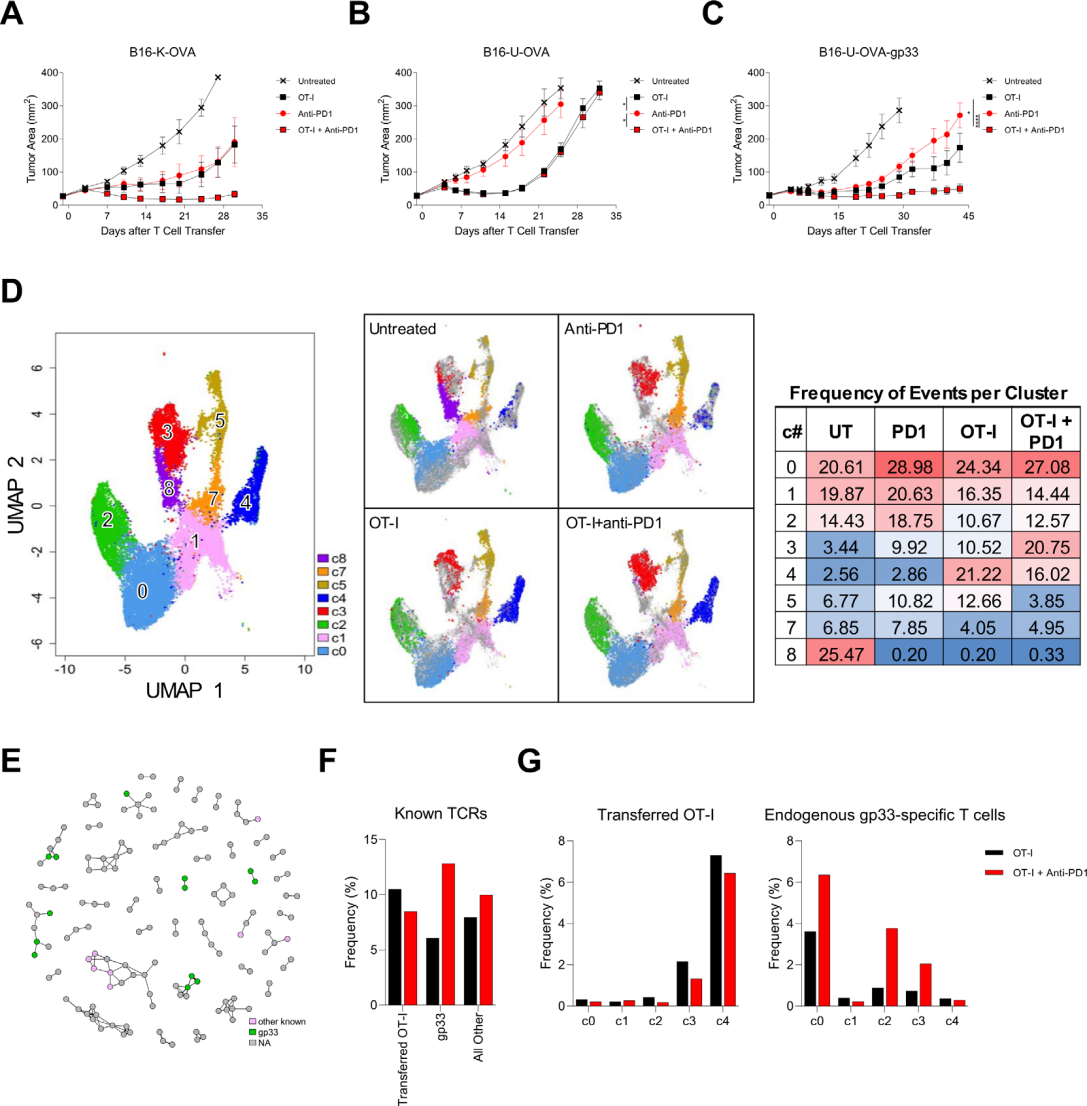
The effect of PD-1 blockade was dependent on tumor antigenicity and endogenous T cell responsiveness to PD-1 blockade. (A) Tumor response to treatment with adoptive T cell therapy with or without PD-1 blockade. C57BL/6J mice with established B16-K-OVA tumors were treated with host conditioning followed by the therapy indicated in the symbol legend. ‘OT-I’ indicates OT-I T cells. ‘Anti-PD-1’ indicated anti-PD-1 antibody. The data are represented as mean±SEM. The data shown have five mice per group and are representative of three independent experiments. (B) Tumor response to treatment with adoptive T-cell therapy with or without PD-1 blockade. C57BL/6J mice with established B16-U-OVA tumors were treated with host conditioning followed by the therapy indicated in the symbol legend. ‘OT-I’ indicates OT-I T cells. ‘Anti-PD-1’ indicated anti-PD-1 antibody. The data are represented as mean±SEM. Statistical significance was determined with a mixed model two-way analysis of variance (ANOVA) with repeated measures and * represents p<0.05. The data shown have five mice per group and are representative of three independent experiments. (C) Tumor response to treatment with adoptive T cell therapy with or without PD-1 blockade. C57BL/6J mice with established B16-U-OVA-gp33 tumors were treated with host conditioning followed by the therapy indicated in the symbol legend. ‘OT-I’ indicates OT-I T cells. ‘Anti-PD-1’ indicated anti-PD-1 antibody. The data are represented as mean±SEM. Statistical significance was determined with a mixed model two-way ANOVA with repeated measures and * represents p<0.05, **** represents p<0.0001. The data shown have five mice per group and are representative of three independent experiments. (D–G) Single-cell RNA and single-cell TCR sequencing data of T cells enriched from OT-I treated C57BL/6J mice with established B16-K-OVA-gp33 tumors. (D) UMAP clusters from all single-cell RNA-sequencing events that contain TCR sequences and exhibit CD3 expression (left). Subsets of the UMAP data for each treatment group: Untreated, Anti-PD-1, OT-I, OT-I + anti-PD-1 (middle). A heatmap of the distribution of events in these clusters for each treatment group (right). (E) Network analysis of TCR specificity groups, including subsets annotated by CDR3β sequences with specificities to defined antigens. Each dot is a TCR specificity group, and edges indicate the presence of identical CDR3β sequence(s) shared across two specificity groups. TCR specificity groups that belong to a community with at least two members are shown. (F) Percentage (frequency %) of TCRβ clonotypes with defined antigen specificities in tumors from OT-I treated and OT-I + anti-PD-1 treated groups. Other than the OT-I TCR, specificities to gp33 or all other (ovalbumin and undefined B16 melanoma tumor antigens) T-cell antigens are determined experimentally and bioinformatically with the GLIPH2 algorithm as in (E). G) Breakdown of cell states for OT-I TCR (left) and TCRβ clonotypes specific to gp33 (right) as defined in (F). PD-1, programmed cell death protein-1; TCR, T-cell receptor; UMAP, Uniform Manifold Approximation Projection.

Given that improvement of T-cell therapy by addition of PD-1 blockade required host endogenous T cells, we postulated that the enhanced efficacy was mediated by endogenous T cell targeting of immunogenic tumor antigens. To explore this possibility, we investigated if improved treatment with PD-1 was abrogated by decreased tumor antigenicity. The B16-K-OVA tumor cell line was engineered to replace mKate2, a foreign protein, with ubiquitin, a self-protein (B16-U-OVA). Addition of PD-1 blockade to cell therapy improved treatment of B16-K-OVA tumors but not B16-U-OVA tumors (figure 5A, B). To test if the benefit of PD-1 blockade was restored by introduction of an immunogenic tumor antigen, B16-U-OVA tumors were engineered to also express gp33 (B16-U-OVA-gp33). Addition of PD-1 blockade increased the antitumor effect of cell therapy in B16-U-OVA-gp33 tumors (figure 5C). Interestingly, while PD-1 blockade alone mediated antitumor activity in B16-K-OVA tumors (figure 5A) but not in B16-U-OVA tumors (figure 5B), addition of the gp33 antigen to B16-U-OVA tumors restored responsiveness to PD-1 blockade alone (figure 5C). These results indicated that tumor immunogenicity and host responsiveness to PD-1 blockade was critical to the effect of PD-1 blockade on the efficacy of cell therapy.

To study the impact of PD-1 blockade on the phenotypic states of OT-I and endogenous T cells, paired TCRα/β sequencing coupled with single-cell RNA sequencing transcriptomics was performed. Tumor-infiltrating T cells were studied 7 days after treatment to ensure tumors of comparable size in each group. The initial UMAP results revealed some clusters enriched in certain treatment groups (figure 5D). Cluster 3 was a population that became enriched when anti-PD-1 was used (figure 5D). Interestingly, genes enriched in this cluster are associated with the antigen presentation pathway and caveolar-mediated endocytosis signaling (online supplemental figure 2A). Cluster 8 was predominantly found in the untreated group and appeared to be bystander T cells as this cluster had no informative genes found to be statistically upregulated compared with any other cluster (figure 5D, online supplemental figure 2A). Additionally, CD4 T cells primarily resided in clusters 5 and 7, with cluster 5 containing the regulatory T cell fraction of this compartment (online supplemental figure 2B). In this assay, we identified OT-I T cells and endogenous gp33-specific T cell clonotypes based on the TCR sequences, enabling a comparison of their cell states measured with single-cell RNA sequencing (figure 5D, F and G and online supplemental figure 2C). Our result showed that tumor-infiltrating OT-I T cells demonstrated little overall change with PD-1 blockade (adjusted p values for clusters 0, 1, 2, 3, and 4 were 1.0, 1.0, 0.36, 0.23, and 0.12, respectively with Fisher’s exact tests; figure 5G and online supplemental figure 2C). In contrast, we observed a significant change in the phenotypic states of endogenous gp33-specific T cells within the tumor, consistent with their functional role in the tumor model (adjusted p values for clusters 0, 1, 2, 3, and 4 were 0.01, 1.25×10^–3^, 6.22×10^–7^, and 5.84×10^–3^, respectively, with Fisher’s exact tests).

We sought to delineate the relative effect of PD-1 blockade on transferred OT-I T cells and on endogenous T cells. To enable identification of tumor-antigen-specific T cells, the TCR α-β chain sequences and corresponding antigen specificities of tumor infiltrating T cells following treatment were determined empirically for a panel of TCRs targeting gp33 and other tumor antigens (B16 tumor antigens, mKate2, and OVA). Experimentally testing all 3216 TCRs identified in these data are not feasible but understanding the frequency of antigen-specific T cells and their transcriptional state is important. Therefore, these empirically-defined TCR sequences were used to identify likely related TCR specificity groups with GLIPH2 (figure 5E). Mice with B16-K-OVA-gp33 tumor were treated with OT-I T cells with or without PD-1 blockade, and the frequencies of tumor infiltrating OT-I T cells and of T cells targeting other tumor antigens were determined by TCR specificity inference with the empirical data. Consistent with the results from B16-K-OVA tumors (figures 1B and 2C), the frequency of transferred OT-I T cells decreased with the addition of PD-1 blockade (figure 5F). In contrast, the frequency of endogenous T cells targeting gp33 and other tumor antigens increased with addition of PD-1 blockade. In summary, these findings supported that PD-1 blockade depends on the endogenous, tumor-specific T cells but not the transferred T cells.

## Discussion

Adoptive T-cell therapy has shown promise in certain solid tumors such as melanoma, synovial cell sarcoma, head and neck cancer, cervical cancer, vulvar cancer, and anal cancer.^3–8^ However, while clinical activity sometimes has been remarkable, improvement is needed. PD-1-based immune checkpoint blockade has proven effective in the treatment of diverse cancers, and combination of PD-1-based treatment with cell therapy is a conspicuous treatment strategy. Nonetheless, the effect and mechanism of action of PD-1 blockade in cell therapy for solid tumors is mostly unknown. And the mechanism by which these treatments modalities interact has implications for the design and development of new treatments.

We found that addition of PD-1 blockade to adoptive T-cell therapy increased tumor regression in solid tumor models. However, several experimental findings indicated that the effect of PD-1 blockade was mediated through endogenous T cells rather than transferred T cells: (1) addition of PD-1 blockade increased tumor infiltration by endogenous T cells but not by transferred T cells, (2) PD-1 blockade did not have a direct effect on T cells for adoptive transfer in the in vitro assays, (3) expression of PD-1 by transferred T cells was not required for PD-1 blockade to augment treatment in vivo, (4) the PD-1 blockade effect required tumor expression of antigens that could be targeted by PD-1-sensitive endogenous T cells, and (5) PD-1 blockade increased tumor infiltration by and caused cell state changes primarily in endogenous rather than transferred antitumor T cells. One implication of these findings is that sequential therapy and combination therapy may be equally effective with transferred cells and PD-1 blockade. Another implication is that *PDCD1* expression by transferred T cells may not limit the efficacy of transferred TCR-T cells, and therefore *PDCD1* disruption or knockdown may not improve treatment.

Other clinical data from small studies also align with this concept. Addition of pembrolizumab to anti-GD2 CAR-T cells did not increase transferred T cell expansion or persistence.^22^ Disruption of *PDCD1* with CRISPR technology in anti-NY-ESO-1 TCR-T cells did not result in clinical responses.^23^ Addition of atezolizumab to anti-CD-19 CAR-T cells for the treatment of diffuse large B cell lymphoma did not increase CAR-T cell persistence or efficacy significantly as compared with historical controls.^25^ In addition, TCR-T cells targeting either E6 or E7 for treatment of metastatic HPV-associated cancers displayed low frequency of PD-1 expression following administration suggesting that most transferred cells were not susceptible to PD-1-mediated inhibition.^6 7^ Finally, in the same studies, PD-1 expression by transferred TCR T cells did not correlate with treatment response.^6 7^ Additionally, when pembrolizumab was given after anti-mesothelin CAR-T cell therapy there was evidence of an additive benefit due to clonal expansion of endogenous T cells.^24^ Taken together, the available clinical data on combination cell transfer and PD-1 blockade therapy conform with the findings in the present study.

The in vivo models employed in this study were designed to replicate the core characteristics of the transferred T-cell therapies that have clinical activity in solid tumors in humans. These characteristics include the use of a TCR rather than a CAR, targeting of a tumor-restricted antigen, transfer of high-avidity T cells, administration of high numbers of T cells, and pretreatment with non-myeloablative host conditioning. Changes in model components such as the parental tumor type, model antigen, model TCR, level of tumor PD-L1 expression, and anti-PD-1 dosing schedule did not change the central finding that endogenous T cells were required for PD-1 blockade to mediate therapeutic benefit. Nonetheless, the results of this study may not apply to other tumor models and clinical settings such as those that target self-antigens, use CAR-T cells, transfer low numbers of T cells, or employ an attenuated conditioning regimen.

## Conclusion

In summary, this study demonstrates, in a range of solid tumor models, that the benefit of PD-1 blockade in adoptive T-cell therapy is driven by an effect on the endogenous T-cell repertoire and the inherent antigenicity of the tumor rather than by an effect on the transferred T cells. These findings have important implications for the design and development of new adoptive T-cell therapy strategies.

## Data Availability

Data are available upon reasonable request. Further information and requests for resources and reagents should be directed to and will be fulfilled by Lead Contact Christian Hinrichs (ch997@cinj.rutgers.edu). The plasmids, cell lines, and data generated in this study will be made available on request.
