# Supplementary figures and images for: Non-synergy of PD-1 blockade with T-cell therapy in solid tumors
Source: J Immunother Cancer. 2022 Jul 6;10(7):e004906. doi: 10.1136/jitc-2022-004906 (PMC9260838; doi:10.1136/jitc-2022-004906)

Supplemental Figure 1

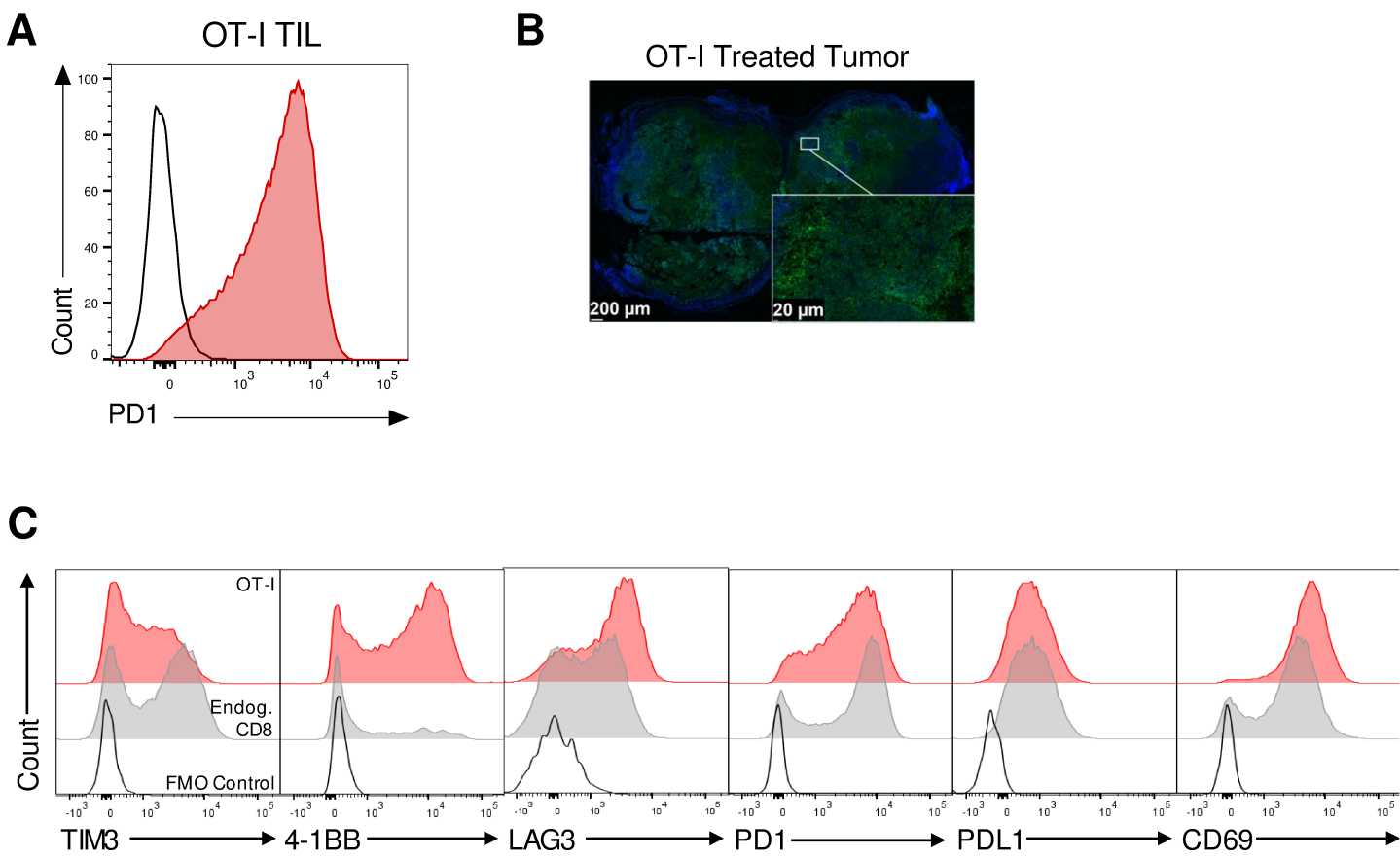

Supplemental Figure 2

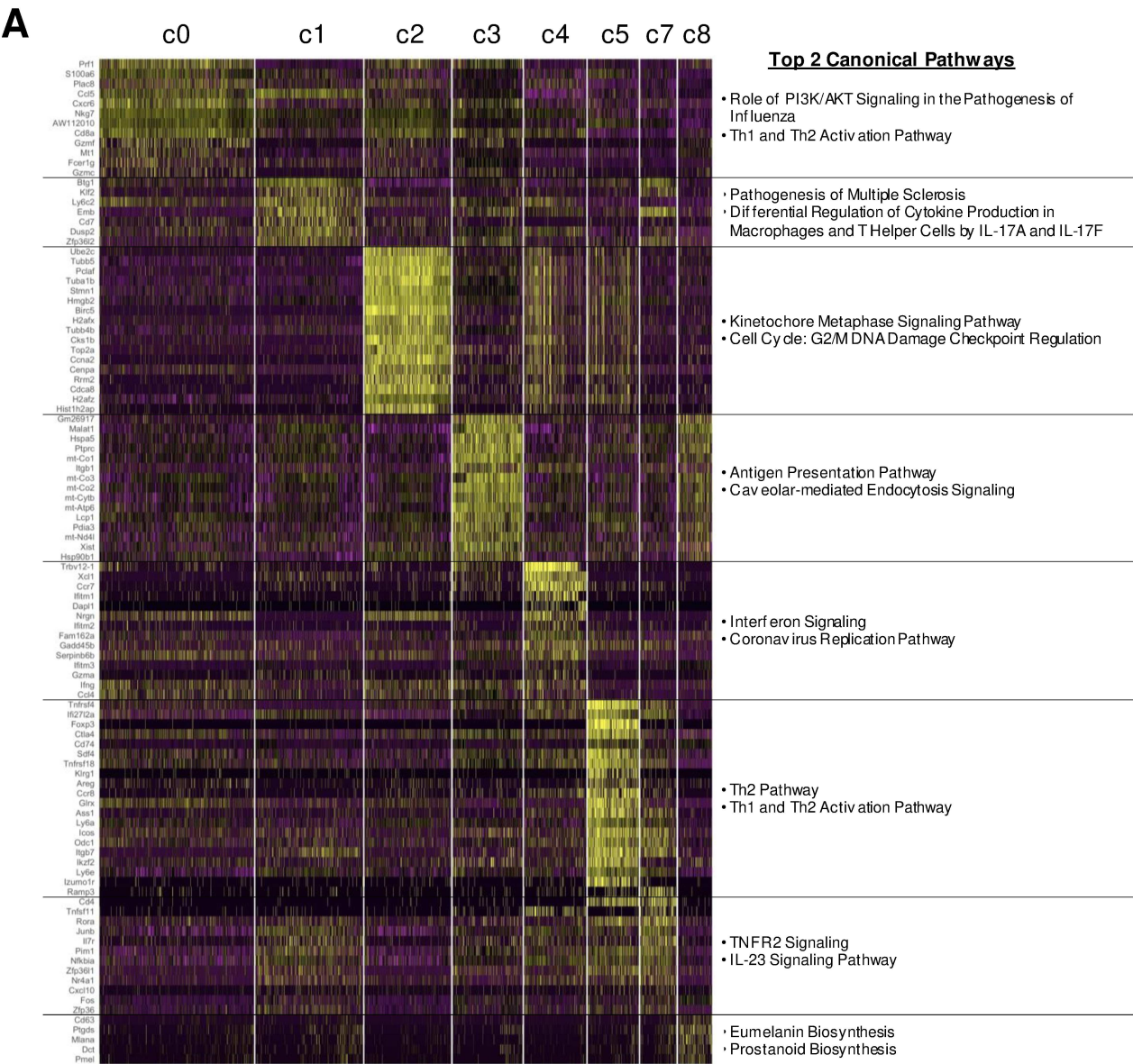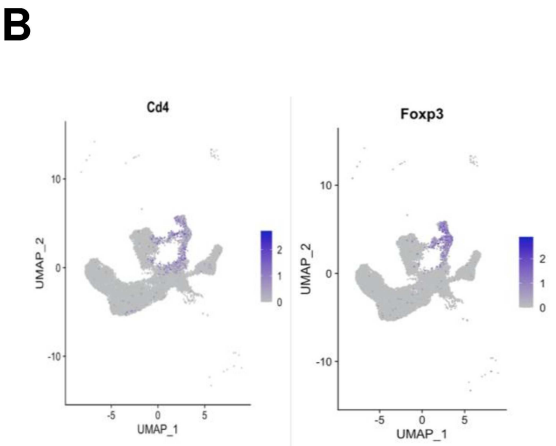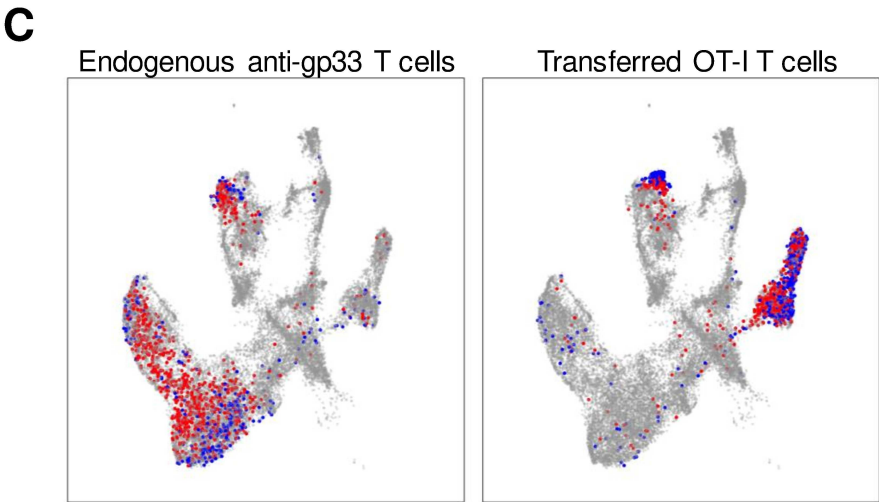

Supplement: Supplementary data [file jitc-2022-004906supp001.pdf]
